# Transvenous interventional implantation of CRT‐D in a critically ill Mustard patient waiting for heart transplant

**DOI:** 10.1002/ccr3.2183

**Published:** 2019-06-14

**Authors:** David Mörtsell, Ulf Thilén, Patrik Tydén, Ole Kongstad

**Affiliations:** ^1^ Cardiology Department Skåne University Hospital Lund Sweden

**Keywords:** cardiac resynchronization therapy, heart failure, intracardiac imaging techniques, transposition of great vessels

## Abstract

Cardiac resynchronization therapy may stabilize patients with severe heart failure awaiting heart transplant. Transvenous interventional implantation aided by intracardiac echocardiography is feasible in patients with adult congenital heart disease.

## 1 INTRODUCTION

Cardiac resynchronization therapy (CRT) in adult congenital heart disease patients is feasible. We describe successful transvenous implantation in a critically ill Mustard patient awaiting heart transplant.

Transvenous implantation of an ICD lead to the subpulmonary ventricle and subsequent puncture and dilatation aided by intracardiac echocardiography to facilitate placement of a pacemaker lead in the systemic ventricle was successful. QRS narrowed from 235 to 160 ms, and cardiac index improved from 1.8 to 3.2 L/min/BSA, which translated into a more stable clinical situation.

Upgrade to cardiac resynchronization therapy (CRT) is indicated in symptomatic chronic heart failure patients with impaired systolic left ventricular function and right ventricular pacing dependency.^1^ The role of CRT in hemodynamically unstable patients awaiting heart transplant is unclear, and reports of feasibility of transvenous CRT implantation in Mustard‐/Senning patients are scarce. We report on the outcome of such a critically ill patient.

## 2 METHODS

**Figure 1:**
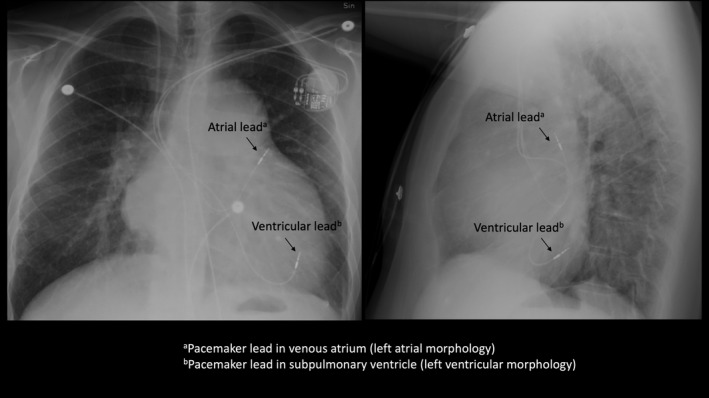
X‐ray after dual chamber pacemaker implant. Anterior‐posterior view (left) and lateral view (right) of lead positions

Male born 1976 with transposition of the great arteries who had Mustard surgery (atrial switch) performed at one year of age. Permanent atrial tachyarrhythmia since 2009 with normal ventricular rate response without any rate‐control medication. Drug history included warfarin, losartan, and spironolactone. The patient presented in September 2017 with symptoms of progressive heart failure, dizziness, and syncope. Echocardiography showed a moderate impairment of the systemic ventricular (right ventricular morphology) systolic function and the ECG a high degree AV‐block with a wide QRS escape rhythm at 40 bpm. He was referred to our department for a dual chamber pacemaker implant. Two leads were advanced through the conduit from the superior vena cava to the systemic venous atrium. The atrial lead was placed posterolaterally in the atrium, and the ventricular lead advanced to the subpulmonary ventricle (left ventricular morphology) and placed in a posterior location (Figure 1).

Tracking of the chronic atrial tachycardia with 1:1 conduction and a ventricular pacing rate of 150 bpm caused hemodynamic shock, but the patient was stabilized after reprogramming the pacemaker to ventricular pacing only (VVIR mode). Although symptoms of dizziness improved, heart failure symptoms progressed rapidly and the patient remained hospitalized in the referring hospital over four months, despite treatment with inotropic agents such as milrinone and levosimendan pulses as well as continuous furosemide infusion to maintain an adequate diuresis. Echocardiography now showed severe systolic dysfunction of the systemic ventricle. The patient was again hospitalized in our department and accepted for a heart transplant and put on the waiting list. An upgrade of his pacemaker system with an epicardial systemic ventricular lead was discussed with the cardiac surgeons, but both epicardial leads and ventricular assist device surgery were judged to be high‐risk procedures given the patients previous cardiac surgery and very poor systemic ventricular and impaired subpulmonary ventricular function. The patient was subsequently accepted for a transvenous upgrade to a cardiac resynchronization therapy‐defibrillator (CRT‐D) system with an endocardial systemic ventricular lead in March 2018. The procedure was performed under local anesthesia and light sedation (low‐dose midazolam). Femoral venous access from the left groin (right femoral vein had been ligated during infancy) included negotiating an S‐curved collateral branch with a microcatheter and a hydrophilic wire into the inferior vena cava, subsequently exchanged to a super‐stiff Amplatz wire facilitating placement of a long sheath in the lower conduit. An intracardiac echocardiography (ICE) catheter was then advanced through the sheath to the systemic venous atrium to guide the surgeon. From the left pectoral infraclavicular region via a percutaneous puncture of the axillary vein, a guidewire was advanced through the conduit into the systemic venous atrium; a long sheath (CPS Direct® SLII straight, St Jude Medical) was then advanced supporting the introduction of a single‐coil implantable cardioverter‐defibrillator (ICD) lead (Durata 7122®, St Jude Medical) to the subpulmonary ventricle. The lead was positioned in a high septal position below the atrio‐ventricular valve. Sensing and threshold were excellent. The pocket was now opened, leads were freed from the tissues, and both preexisting atrial and ventricular pacemaker leads were extracted with simple traction. Continuous pacing via the ICD lead was necessary since the patient now demonstrated total pacing dependency. A second puncture of the axillary vein followed and a guidewire was advanced to the superior vena cava “pouch” and conduit adjacent to the systemic venous atrium. A short deflectable sheath (Agilis®, St Jude) was then advanced to the conduit and a radiofrequency guidewire (Nykanen RF Wire®, Bayliss Medical) through this. Guided by ICE, the surgeon directed the deflectable sheath in an inferior‐anterior angle toward the pulmonary venous atrium, and after 2 seconds of RF energy applied, the guidewire could enter the pulmonary venous atrium followed by the sheath. The guidewire was then exchanged for a standard 0.035” wire, and the sheath advanced to the systemic ventricular apex. The sheath was now exchanged to a CRT delivery sheath (CPS Direct® SLII RS, St Jude Medical), and the sheath positioned below the atrio‐ventricular valve against the basal anterolateral portion of the systemic ventricle. A pacemaker lead (SelectSecure® 3830, Medtronic) was then advanced and positioned here, and the sheath was removed. Both leads were then connected to a CRT‐D device (Unify Assura®, St Jude Medical), and the pocket was closed. An antibacterial envelope (TYRX®, Medtronic) was used to reduce the risk of infection. A hemodynamic study was performed the day before the procedure and again the day after, as well as a transthoracic echo.

## 3 RESULTS

**Figure 2:**
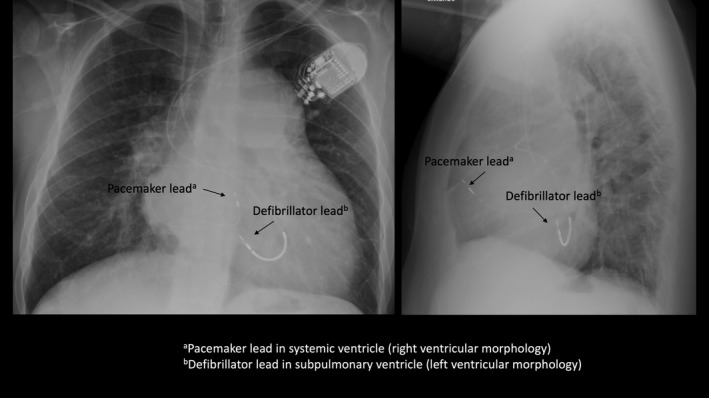
X‐ray after upgrade to CRT‐D. Anterior‐posterior view (left) and lateral view (right) of lead positions

**Figure 3:**
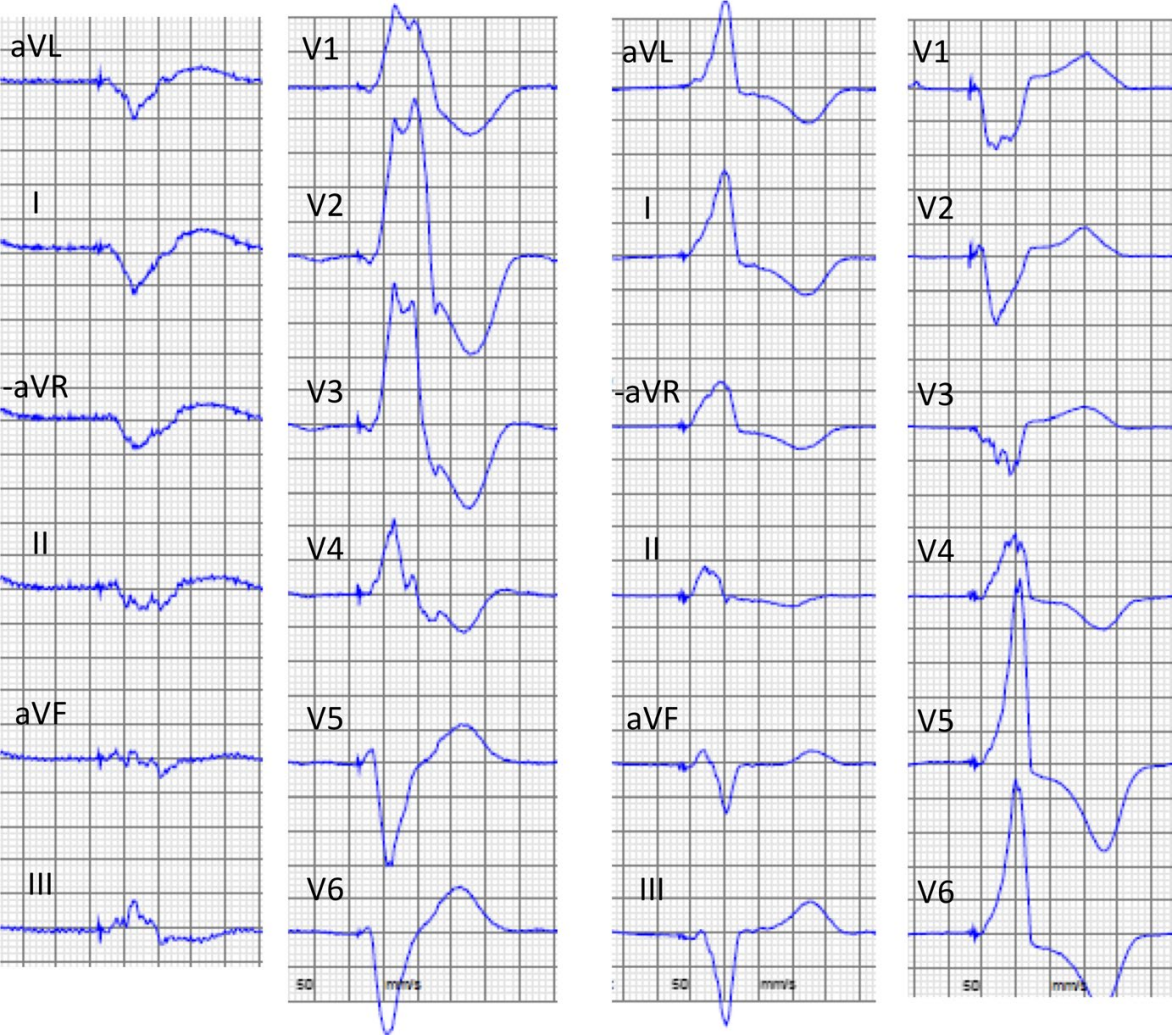
ECG before and after upgrade to CRT. Left panel showing pacing before upgrade (RBBB pattern, QRS width 235 ms) and right panel showing CRT‐pacing (QRS width 160 ms)

Improved hemodynamic and echocardiographic measurements (Table 1) translated into a clinical situation where all inotropic agents and the continuous furosemide infusion could be stopped. Systemic blood pressure was now over 100 mm Hg for the first time in 6 months, and the patient could now be mobilized and move about freely in the ward. An x‐ray was performed after 2 weeks (Figure 2) and showed stable lead positions and technical parameters remained excellent with pacing thresholds <0.5 V on both leads. The paced QRS width of the ECG was now much shorter than previously, from 235 ms (left ventricular pacing) to 160 ms (CRT) (Figure 3).

**Table 1:** Acute hemodynamic effects of CRT

	PACEMAKER	CRT
CI (L/min/BSA)	1.8	3.2
SV (mL)	45	80
SVO2 (%)	48	67
EF (%)	10	25

Note

Swan‐Ganz catheterization and echocardiography during pacing, rate 70 beats per minute.

The remarkable acute hemodynamic effects of biventricular pacing did not reverse the patient's condition, but rather stabilized the patient and thus made it possible avoid an “urgent call” transplant. The patient subsequently underwent a successful heart transplant three months after CRT upgrade and is now doing well.

## 4 DISCUSSION

We describe a successful case of transvenous CRT in a critically ill Mustard patient with a failing systemic ventricle awaiting heart transplant. Mustard patients frequently have mechanical dyssynchrony in the systemic ventricle,^2^ and acute temporary biventricular pacing improves the systemic ventricular contractility.^3^ Transvenous CRT in a Mustard patient has been reported,^4^ but the feasibility of CRT in hemodynamically unstable severe heart failure Mustard‐/Senning patients awaiting heart transplant is unclear. In the ordinary heart transplant patient with a failing left ventricle, implantation of a left ventricular assist device as a bridge to transplant is the recommended therapy. However, in patients with adult congenital heart disease (ACHD), a failing systemic ventricle and a history of repeated sternotomies, ventricular assist surgery may be difficult or contraindicated and other therapies can be explored.

## 5 CONCLUSION

Upgrade to CRT may alleviate symptoms of severe heart failure and reduce the need of inotropic agents and hospitalization in selected ACHD‐patients with advanced heart failure awaiting heart transplant. Interventional techniques and perioperative imaging make transvenous CRT‐D implantation feasible in patients having had a Mustard repair.

## CONFLICT OF INTEREST

There are no relevant financial disclosures for this manuscript.

## AUTHOR CONTRIBUTION

DM: served as surgeon, designed the procedure, and drafted and revised the manuscript. UT: carried out the hemodynamics assessment, followed up the patients, revised the manuscript, and provided funding of publication fees. PT: carried out the clinical assessment during in‐hospital treatment, followed up the patients, and revised the manuscript. OK: served as assisting surgeon (including intracardiac echocardiography), designed the procedure, and revised the manuscript.
